# Prevalence and Correlates of Night Eating in the German General Population

**DOI:** 10.1371/journal.pone.0097667

**Published:** 2014-05-14

**Authors:** Martina de Zwaan, Astrid Müller, Kelly C. Allison, Elmar Brähler, Anja Hilbert

**Affiliations:** 1 Department of Psychosomatic Medicine and Psychotherapy, Hannover Medical School, Hannover, Germany; 2 Center of Weight and Eating Disorders, Perelman School of Medicine, University of Pennsylvania, Philadelphia, Pennsylvania, United States of America; 3 Department of Medical Psychology and Medical Sociology, University of Leipzig Medical Center, Leipzig, Germany, and Department of Psychosomatic Medicine and Psychotherapy, University Medical Center Mainz, Mainz, Germany; 4 Integrated Research and Treatment Center Adiposity Diseases, Department of Medical Psychology and Medical Sociology, University of Leipzig Medical Center, Leipzig, Germany; Technion - Israel Institute of Technology, Israel

## Abstract

Recently, night eating syndrome (NES) was included into the DSM-5 as an example of “Other Specified Feeding and Eating Disorders.” The study provides insight into the population prevalence of NES using a large representative German population sample (n = 2,460) with a wide age range (14–85 years). The prevalence of NES was 1.1% using a cut-off on the Night Eating Questionnaire (NEQ) of 25. A positive screening for NES was positively associated with depression and anxiety, eating disorder psychopathology, and body weight.

## Introduction

The night eating syndrome (NES) was first described by Stunkard et al. in 1955 [1] among obese individuals characterized by unsuccessful weight management. It was conceptualized as a combination of eating disorder, sleep disorder, and mood disorder [1]. Since then, the definition of NES has varied. Recently, NES was included in the DSM-5 as an “Other Specified Feeding and Eating Disorder” characterized by “recurrent episodes of night eating, as manifested by eating after awakening from sleep or by excessive food consumption after the evening meal. Awareness and recall of the eating is required, as are significant distress or impairment caused by the disorder. Exclusion criteria are binge-eating disorder or another mental disorder, as well as medical disorders or medication that might better explain the disordered eating pattern” [2]. Inclusion in the DSM-5 was predated by the diagnostic criteria developed in the First International Night Eating Symposium [3]. In addition, the Night Eating Questionnaire (NEQ) was developed and validated as a screening tool and severity measure for the NES [4].

The prevalence of the NES is estimated at 1.5% in the general U.S. population [5] and 1.6% in a U.S. community sample of young women [6]. A Swedish study reported a prevalence of broadly defined night eating of 4.3% in men and 3.4% in women using a large population-based sample of Swedish twins [7]. NES is more prevalent (up to 50%) in selected clinical populations with obesity and diabetes [8]. The prevalence is also high among patients with various psychiatric disorders (12–35%) [9], [10]. NES appears to be associated with obesity and psychiatric co-morbidity, e.g. lifetime mood and anxiety disorders. Studies suggest some overlap between NES and other eating disorder syndromes such as binge eating disorder [7], [11]. There is now evidence that antidepressants (selective serotonin reuptake inhibitors) and cognitive behavioral therapy may be effective treatment approaches for NES [12].

The present study aimed to estimate the prevalence and correlates of NES in a large representative German sample using a validated self-report measure (NEQ) as the screening tool.

## Methods

### Ethics Statement

All participants were visited in-person and informed about the study procedures by a trained research assistant. They also received a written explanation of the study including information on data management and provided their oral informed consent prior to assessment (for minor participants, oral informed consent was also obtained from one parent). Oral consent is common in survey research in Germany. The ethical guidelines of the International Code of Marketing and Social Research Practise by the International Chamber of Commerce and the European Society for Opinion and Marketing Research were followed. The Ethics Committee of the University of Leipzig Medical School approved the study including the consent procedure.

### Participants

A representative sample of the German general population was selected with the assistance of a demographic consulting company. The sample was selected to be representative in terms of age, sex and education. A total of 2,508 people aged between 14 and 92 years agreed to participate and completed the self-rating questionnaires (participation rate: 58% of valid addresses) between April 20 and June 16, 2013. Participants who did not fully complete the instruments and those who did not provide weight data were excluded from further analyses (n = 52), yielding a final sample of 2,456 individuals.

### Instruments

The German version of the NEQ, the only validated screening tool for NES was used [4], [13]. The NEQ is a 14-item self-report inventory assessing the behavioral and psychological symptoms of NES including nocturnal ingestions, evening hyperphagia, morning anorexia, and mood/sleep. The answers are given on a five point Likert-type scale (0–4) with a NEQ total score ranging from 0 to 52 points. A total score of ≥25 indicates that a person likely has difficulty with NES.

The 4-item Patient Health Questionnaire (PHQ-4) is an ultra-brief self-report questionnaire for use as an overall screening tool for depression and anxiety [14], [15]. Since the subscales PHQ-2 (depression) and GAD-2 (anxiety) are highly inter-correlated (r = 0.61) the authors considered the PHQ-4 total scale as an overall screening tool for depression and anxiety using a cut-off of ≥6. The PHQ-4 total score ranges from 0 to 12 points.

Finally, a newly developed short version of the Eating Disorder Examination-Questionnaire [16], [17], the EDE-Q8 [Hilbert et al. – unpublished data] was used, assessing eating disorder psychopathology (0, ‘not at all’ to 6, ‘extremely’).

Based on the participants’ self-reported height and weight, BMI (kg/m^2^) was calculated.

### Statistics

Statistical analyses were conducted using PASW 18.0.0 for Windows. The study data were weighted for age, sex, and state of residency according to the distribution of these socio-demographic factors in the German adult population as given by the Federal Statistics Office to ensure representativeness leading to a sample size of 2,460 participants. Associations between NEQ and other variables were determined using chi square tests and t-tests as appropriate. A logistic regression analysis was conducted with NES as the dependent variable and age, BMI, PHQ-4 scores, and EDE-Q8 scores as independent variables. An alpha-level of 0.05 was adopted for all tests.

## Results

The mean age of the weighted sample was 48.1 years (SD 19.0 years), with 51.1% being female (n = 1,256). 52.3% (n = 1,286) were married, 4.7% (n = 116) were unemployed, and 21.7% (n = 533) had finished high school or attained education beyond high school. A total of 1.1% (n = 27) of the sample screened positive for NES with a total NEQ score of ≥25. For the two core NES criteria, 6.2% (n = 153) of the full sample reported consuming more than 25% of their daily caloric intake after dinner and 1.3% (n = 32) reported eating during at least half of their awakenings. Screening for current depression and anxiety with the PHQ-4 revealed positive results in 7.3% (n = 180) of the sample.

No associations between a positive NES screening result and any of the socioeconomic parameters (age, sex, marital status, education, employment status, area of living) were found. Significant positive associations emerged between a positive NES screening and BMI, level of anxiety/depression (PHQ-4 scores), and eating disorder psychopathology (EDE-Q8 scores) (Table 1). Age-adjusted binary logistic regression analysis confirmed these results showing that BMI (OR 1.126, 95% CI 1.040–1.220), PHQ-4 (OR 1.356, 95% CI 1.322–1.705), and EDE-Q8 scores (OR 1.502, 95% CI 1.068–1.721) were independently associated with a positive NES screening result.

**Table 1:** Comparison between participants with and without a positive screening result for NES (weighted sample).

Variables Mean (SD)	NES positive N = 27	NES negative N = 2,432	Statistics, t (df), p	Effect Size*
Age, years	41.9 (17.4)	48.2 (19.0)	1.709 (2458), .088	–
BMI, kg/m^2^	28.4 (6.8)	25.1 (3.7)	−2.531 (26,362), .018	0.88
PHQ-4 score	5.6 (2.8)	1.6 (2.2)	−7.152 (26,532), <.001	1.81
EDE-Q8 score	2.8 (1.9)	0.9 (1.3)	−5.033 (26,453), <.001	1.45

BMI: body mass index, PHQ-4: Patient Health Questionnaire-4 item version; EDE-Q8: Eating Disorder Examination Questionnaire-8 item version.

*Cohen’s *d*: 0.2 small, 0.5 medium, 0.8 large effect.

## Discussion

The present study is the first to investigate the occurrence of NES in a European country using the NEQ which is the only well-recognized self-report measure for NES screening [4]. The prevalence of NES was estimated at 1.1% in a large scale representative sample of the German population, which is comparable to other population-based samples [5], [6]. We did not find a gender or age difference between participants with or without a positive NES screening result. In line with previous reports, our findings indicate a strong link between NES and BMI, eating disorder psychopathology and anxiety and depressive symptoms. Many but not all studies have observed a positive relationship between NES and body weight, with some evidence that NES may lead to weight gain over time [8], [11]. In addition, there is evidence that NES might impair weight loss, weight loss maintenance, and even diabetes management [12] supporting the clinical relevance of NES. An association between depression and NES has been reported in numerous studies in different samples; however, it remains unclear whether depressed mood is a cause, consequence, or clinical feature of NES. This co-morbidity suggests that individuals with NES should be assessed for co-morbid mental disorders.

The study is nevertheless limited by the use of a self-report screening instrument to determine NES and no additional information on medical co-morbidities or medication intake that might influence sleep and hunger was available. The proposed research diagnostic criteria for NES constitute a basis for the development of valid diagnostic tools and more systematic inquiry, with treatment of the syndrome still in its infancy [18]. However, the criteria’s utility, both in research and clinical practice, remains to be tested.

## Supporting Information

Study Data S1 (SAV)
